# High‐Impulse, Modular, 3D‐Printed CubeSat Electrospray Thrusters Throttleable via Pressure and Voltage Control

**DOI:** 10.1002/advs.202413706

**Published:** 2025-02-11

**Authors:** Hyeonseok Kim, Luis Fernando Velásquez‐García

**Affiliations:** ^1^ Department of Mechanical Engineering Massachusetts Institute of Technology 77 Massachusetts Ave Cambridge MA 02139 USA; ^2^ Microsystems Technology Laboratories Massachusetts Institute of Technology 77 Massachusetts Ave Cambridge MA 02139 USA

**Keywords:** 3D‐printed CubeSat hardware, electric space propulsion, electrospray, space technology

## Abstract

This study reports the proof‐of‐concept demonstration of novel, additively manufactured, droplet‐emitting electrospray emitter arrays for CubeSat thruster applications. The modular thruster design incorporates multiscale features by employing two different vat photopolymerization technologies, i.e., digital light processing for defining mesoscale features, and two‐photon polymerization for creating microscale features. The thruster design includes optimized, 50 µm‐diameter microfluidic channels to attain uniform emitter array operation. Devices with up to 8 modules of 4 emitters were tested in a vacuum to assess their performance. Stable and uniform electrospray emission was achieved across all emitters, with a near 100% transmission across the extractor. Both pressure (flow rate) and voltage modulation are investigated as methods for controlling the emitted current and, by extension, the thrust generated by the devices. The per‐emitter current followed a well‐known square root relationship with flow rate; in addition, a linear relationship between per‐emitter current and extractor voltage is observed. Compared to pressure control, modulating thrust via voltage control simplifies system design, eliminating the need for complex valves and enabling a wider throttle range. Estimated thrust and specific impulse are comparable to, or better than reported droplet‐emitting electrospray thrusters. These findings demonstrate the potential of additive manufacturing to implement electrospray propulsion hardware.

## Graphical abstract

This study reports the proof‐of‐concept demonstration of novel, additively manufactured, droplet‐emitting electrospray emitter arrays for CubeSat thruster applications. The modular thruster design incorporates multiscale features that enable scaling up thrust with low power consumption by employing two different vat photopolymerization technologies, i.e., digital light processing for defining mesoscale features and two‐photon polymerization for creating microscale features.

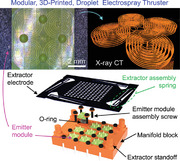

## 1 Introduction

Electrospray propulsion is achieved by applying a high electric field to the free surface of a conductive liquid, causing the electrohydrodynamic ejection of charged particles.^[^ ^1^ ^]^ This process is triggered when the liquid meniscus deforms into a Taylor cone under sufficient electric stress, which balances electrical and surface tension forces.^[^ ^2^ ^]^ At the apex of the cone, high acceleration enhances convection, sometimes to the point where the liquid surface can no longer shield the external electric field. This induces a transition in the flow structure into a thin jet that breaks up into droplets, where the dominant charge transport mechanism shifts from conduction to convection.^[^ ^3^, ^4^, ^5^ ^]^ The resulting cone‐jet mode in electrospray is particularly valuable for uniformly generating droplets and ions in a controlled manner, making it useful in applications such as mass spectrometry,^[^ ^6^ ^]^ material synthesis,^[^ ^7^, ^8^ ^]^ deposition technology,^[^ ^9^ ^]^ and spacecraft propulsion.

Electrospray propulsion offers significant advantages for small spacecraft like CubeSats, where efficient use of onboard propellant is critical. While chemical rockets are effective for launch, they become inefficient in orbit due to the large amount of propellant required for maneuvers. In contrast, electrospray thrusters are not constrained by the energy per unit of flow rate released from chemical reactions and are better suited for long‐duration, low‐thrust operations, making them ideal for orbital adjustments.^[^ ^10^ ^]^ Furthermore, the performance of an electrospray system improves with miniaturization, as lower voltages are required to trigger electrospray emission from smaller emitters. Electrospray thrusters can also operate in bipolar mode, eliminating the need for a neutralizer, which could consume additional propellant, even at a level comparable to that directly employed in producing thrust.^[^ ^11^ ^]^

Electrospray thrusters are versatile, offering a wide range of thrust and specific impulse depending on the mode of operation. A high specific impulse of thousands of seconds can be achieved through the emission of pure ions, primarily due to their high charge‐to‐mass ratio,^[^ ^12^, ^13^, ^14^, ^15^ ^]^ However, this mode results in relatively low thrust, in the range of tens to hundreds of nanonewtons per emitter, because of the low flow rate associated with attaining ion emission. Conversely, droplet emission provides an order of magnitude higher thrust due to a higher flow rate, at the cost of a decreased specific impulse, typically in the hundreds of seconds range.^[^ ^16^, ^17^, ^18^, ^19^, ^20^ ^]^ Further increase of the thrust delivered by electrospray thrusters can be attained via *multiplexing* the emitters, i.e. implementing an array of emitters that is uniformly operated in parallel.

The fabrication of multiplexed electrospray thrusters has traditionally been conducted in semiconductor cleanrooms to achieve precise microscale features.^[^ ^1^, ^17^, ^20^, ^21^ ^]^ However, these manufacturing processes involve numerous individual steps such as photolithography, thin‐film deposition, and etching, each of which is time‐consuming and expensive. In contrast, 3D printing offers cheaper and faster fabrication of monolithic, complex structures, enabling faster iteration loops. Additionally, 3D printing is compatible with in‐space manufacturing, which adds further value. A current limitation of 3D printing is its resolution, which can hinder the fabrication of small features. Another challenge lies in the fact that electrospray engines are multiscale systems that require the definition of both micro and mesoscale features. Typically, there is a trade‐off between how small features can be defined and how much time it takes to create larger features.

There are reports of massively multiplexed, uniformly operating ion‐emitting electrospray thruster arrays made via 3D printing.^[^ ^15^ ^]^ However, implementing 3D‐printed droplet‐emitting thrusters remains challenging. Unlike ion‐emitting thrusters, which often rely on the external wetting of the emitter of a porous bulk to transport propellant to the emission sites, droplet‐emitting devices require long and narrow capillary emitters with an integrated flow distributor to ensure uniform operation. Non‐uniform flow distribution can lead to efficiency degradation or emitter flooding, which limits the thruster's lifespan.^[^ ^22^ ^]^ Fabricating these high impedance hydraulic structures is a significant challenge beyond just 3D printing. For example, microbeads have been inserted into capillary channels to increase their hydraulic resistance and promote uniform flow across the emitter array, but only 60% to 80% of the emitters in a 91‐emitter device were active.^[^ ^19^ ^]^ Other studies have employed emitters with inner diameters as small as 8 µm^[^ ^23^ ^]^ or microfluidic channels with cross‐sectional areas of 20 by 20 µm,^[^ ^17^, ^18^ ^]^ to achieve uniform operation, but fabricating such small features remains difficult. While 3D‐printed electrospray thrusters with capillary emitters made via two‐photon lithography have been reported, they don't attain steady operation.^[^ ^24^, ^25^ ^]^

This study presents a proof‐of‐concept demonstration of a 3D‐printed, multiplexed, droplet‐emitting electrospray thruster design that attains stable and uniform emission using the ionic liquid 1‐ethyl‐3‐methylimidazolium tetrafluoroborate (EMI‐BF_4_) as a propellant. Devices with as many as 32 emitters uniformly operating in parallel were demonstrated. The reported thruster design is modular, utilizing two different vat photopolymerization techniques to incorporate the multiscale features required to scale up thrust while maintaining low power consumption; specifically, two‐photon polymerization (2PP) is used for creating emitter modules with microscale features that trigger droplet emission and regulate propellant flow, while digital light processing (DLP) is employed for defining mesoscale features to integrate the emitter modules to a common propellant feed and extractor electrode. The microfluidic channels were optimized for uniform flow distribution by enhancing their tolerance to fabrication errors and enabling them to withstand dynamic outlet conditions at the emitter tips caused by non‐uniform electric fields and interfacial effects. Characterization results show that thrust level can be controlled by adjusting either pressure (pressure‐fed flow rate) or voltage. Notably, voltage control showed great promise, offering a wider range of thrust compared to flow rate control, while requiring significantly simpler hardware. The thrust and specific impulse estimates of our thruster design are comparable to, or better than, those of droplet‐emitting electrospray thrusters fabricated via subtractive manufacturing and microfabrication techniques. Section 2 details the design and rationale behind the device, which comprises the manifold block, emitter modules, and extractor electrode. Section 3 describes the fabrication of the devices, while Section 4 presents the results of the electrospray experiments. Section 5 provides a discussion of the experimental results. Finally, Section 6 summarizes the work and provides directions for future research.

## 2 Device Design

**Figure 1:**
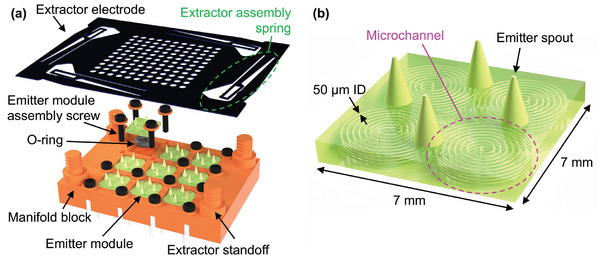
3D CAD models of the electrospray thruster design explored in this study: a) exploded view of assembled thruster made of a manifold block, an array of emitter modules, and an extractor electrode (during the experiments, the central emitter module was replaced with a non‐functional module to ensure uniform emitter operation across the other eight emitter modules); b) close‐up of an emitter module composed of an array of four emitters, each fed by a long, narrow, and winding capillary.

The electrospray thruster design consists of three primary components: the manifold block, the emitter modules, and the extractor electrode (**Figure** **1**). The manifold block supplies propellant to an array of emitter modules from a single inlet. Each emitter module contains four emitters, each of them fed by a different capillary. The modular design of the thruster employs two distinct 3D printing technologies to integrate the multiscale features critical for effective operation. The manifold block was 3D‐printed via DLP to define the mesoscale features required to scale up the number of emitters and accommodate the array of emitter modules. In contrast, the emitter modules were 3D‐printed via 2PP to define the finely featured emitters and capillaries with high precision. Key advantages of the modular design are the ease of repairing the thruster by replacing problematic emitter modules and the flexibility to adjust the number of emitters by replacing selected emitter blocks with non‐functional blocks that do not have open channels. The extractor electrode is a laser‐cut plate made of 1095 steel. The extractor features multiple apertures, each of which aligns with a different emitter, allowing for the transmission with a low interception of the emitted droplets from the Taylor cones to generate thrust.

### 2.1 Manifold Block

The Manifold block is a rectangular prismatic structure measuring 42 mm by 29 mm by 4 mm. The manifold block contains nine pockets to accommodate the emitter modules; the pockets have grooves to install O‐rings to ensure proper sealing. The emitter modules are assembled to the manifold block using screws. Instead of using branching channels to direct propellant flow into each emitter module, the manifold block features a central reservoir between the inlet and the emitter module slots to reduce potential fabrication errors that could affect flow rate variation across the emitter modules. The ceiling of the propellant reservoir is supported by an array of circular pillars. Additionally, four posts at each corner of the manifold block are partially threaded to enable the integration of the extractor electrode using custom 3D‐printed caps. The height of these posts sets the distance between the emitter and extractor, which needs to be in the correct range for successful electrospray operation. The voltage required for electrospray initiation is related to this distance, with the minimum voltage *V*_start_ predicted by the reduced‐order model as:

(1) $$V_{\mathrm{start}}=\sqrt{\frac{\gamma D_{o}}{\varepsilon_{0}}}\;\ln\left[\frac{4G}{\sqrt{D_{o}D_{i}}}\right]$$

where *γ* is the surface tension of the liquid, *D_o_* and *D_i_* are the outer and inner diameters of the emitter, respectively, ε_0_ is the electrical permittivity of vacuum, *G* is the emitter‐extractor separation, and $4G\gg \sqrt{D_{o}D_{i}}.$ ^[^ ^1^ ^]^ Consequently, smaller emitters require lower voltage to trigger electrospray emission, making them more efficient for generating thrust with reduced power consumption. The experimental apparatus used to characterize the devices explored in this study can bias voltages in the ±3000 V range (see Section 4); with outer and inner emitter diameters equal to 150 and 50 µm, respectively, the emitter‐extractor separation should be at most 400 µm, which was validated through extensive electrospray testing.

Despite the design features implemented to attain uniform flow across the array of emitter modules, experiments conducted early on in this study showed that the flow rate to the module at the center of the array was substantially higher than the others, causing operational problems; consequently, the center module was replaced with a non‐functional module during the electrospray tests.

### 2.2 Emitter Modules

The emitter modules are 7 mm by 7 mm by 1.1 mm rectangular prismatic components. Each emitter module contains four conically shaped, evenly spread emitters in a diamond pattern. The inlet channel of each emitter module bifurcates near the inlet and further divides into four branches, each supplying propellant to a different emitter. Via experimentation, it was determined that incorporating rounded transitions into the branch design is critical for achieving uniform flow distribution, even at low Reynolds numbers smaller than unity. Each microchannel is 18.3 cm long with a constant diameter equal to 50 µm. For laminar flow, the hydraulic resistance of a circular channel *R*_h_ can be estimated by:

(2) $$R_{h}=\frac{8\mu L}{\pi r^{4}}\;$$

where *µ* is the viscosity of the liquid, and *L* and *r* are the length and radius of the channel, respectively. Using the propellant EMI‐BF_4_, the hydraulic resistance of each channel is estimated at 3.75 × 10^16^ kg m^−4^ s^−1^; this high hydraulic resistance ensures even propellant distribution to each emitter, as viscous dissipation along the channels must dominate flow dynamics due to the following reason.

The forces acting on the flow fed to each emitter can be divided into three categories: 1) pressure at the manifold, 2) viscous dissipation along the channel downstream from the manifold, and 3) interfacial forces at the liquid‐vacuum interface at the emitter tip. While pressure and viscous forces can be controlled by adjusting the channel geometry and the inlet pressure to the device, interfacial forces, which include surface tension and electrostatic forces, are harder to predict due to fluctuations in the Taylor cone and possible non‐uniform electric fields between emitters. By ensuring that the manifold pressure significantly outweighs electrostatic and capillary forces, the influence of interfacial forces can be minimized, leading to a more uniform flow. Therefore, longer channels with a high hydraulic resistance area are advantageous for uniform operation, though channel length is ultimately constrained by the volume of the emitter module.

However, if the channels are too small, fabrication spread can lead to huge variations in hydraulic resistance between them. To determine the optimal channel diameter, a worst‐case scenario model was proposed for a fixed hydraulic design in which the flow rate variation between two arbitrary emitters was maximized and the channel radius that gives the least flow rate variation in the worst‐case scenario was found. This optimization process, supported by testing with fabricated parts, identified 50 µm as the ideal channel diameter to minimize flow rate discrepancies given the capabilities of the printer used to create the emitter modules via 2PP. A detailed modeling of this optimization process is provided in the appendix.

### 2.3 Extractor Electrode

The extractor electrode is a laser‐cut 1095 steel sheet 243 µm thick. The extractor contains an array of apertures aligned with the emitters affixed in the manifold block to allow the transmission of the electrosprayed droplets. The center‐to‐center separation between adjacent apertures is 2.15 mm, and these apertures are concentric with the emitter tips. Four spring structures were integrated into the extractor electrode to facilitate the assembly of the extractor to the manifold block while ensuring proper in‐plane alignment between the array of apertures in the extractor and the array of emitters affixed to the manifold block.^[^ ^26^, ^27^ ^]^

## 3 Device Fabrication

Both the manifold block and the emitter modules were fabricated using vat photopolymerization, although different 3D printing technologies were employed. The manifold blocks were 3D printed using an Asiga MAX X27 DLP printer (Asiga, Alexandria, Australia) that has a 1920 by 1080 pixels light engine with 27 µm pixel size. The printing feedstock used was SolusProto (Junction 3D, Santa Clarita, CA, USA), enhanced with the photo absorber 2‐nitrophenyl phenyl sulfide (TCI America, Portland, OR, USA) to improve vertical resolution.^[^ ^28^ ^]^ A DLP printer polymerizes each layer in a single projection by adjusting the angles of micromirror arrays corresponding to each pixel, making it suitable for the rapid printing of mesoscale parts. Printing the manifold block with an Asiga MAX X27 took ≈2 h.

The emitter modules were 3D printed with a NanoOne 1000 2PP printer (UpNano, Vienna, Austria). This printer employs a 780 nm, fs‐pulsed laser to selectively polymerize the resin within a small focal volume (voxel). The laser scans line by line to fill‐in the unit area corresponding to the field of view. The stage is then shifted horizontally to cover different locations, and the process is repeated layer by layer to build the structure. To expedite the printing process, the coarse mode was used with a 10X objective lens, employing a layer height of 5 µm, an in‐plane line distance of 4.2 µm, a laser power of 80 mW, and an infill speed of 600 mm s^−1^. In coarse mode, the voxel width in the horizontal direction is widened, enabling faster printing without compromising vertical resolution.^[^ ^29^ ^]^ These settings allowed each emitter module to be printed in ≈3 h using the UpPhoto resin (UpNano, Vienna, Austria). Additionally, the emitter modules were printed in voxel mode, where the part is discretized into voxels and only the voxels fully occupied by the part are printed. This under‐printing approach ensures easier cleaning of the channels by avoiding overly small channel sizes. It is important to point out that the DLP printer used to make manifold block is not capable of making the small features present in the emitter modules; instead, it can reliably make features an order of magnitude larger.

Due to the long‐term exposure of the devices to the propellant EMI‐BF_4_, the durability and chemical stability of the printing feedstock are critical. To assess material compatibility, surface roughness was measured before and after prolonged exposure to EMI‐BF_4_ on small flat‐surfaced printed samples using a laser scanning confocal microscope Keyence VK‐X250 (Keyence, Itasca, IL, USA). The arithmetic average values, shown in **Tables** **1** and **2**, indicate no significant changes over two months, demonstrating the material's resistance to degradation from EMI‐BF_4_ exposure.

**Table 1:** EMI‐BF_4_ compatibility of Solus Proto DLP resin.

Exposure Time [weeks]	Initial surface roughness [µm]	Altered surface roughness [µm]
1	2.673	4.382
2	2.498	2.210
3	2.654	3.716
4	1.912	3.189

**Table 2:** EMI‐BF_4_ compatibility of UpPhoto 2PP resin.

Exposure Time [weeks]	Initial surface roughness [µm]	Altered surface roughness [µm]
1	1.803	1.894
2	2.097	1.932
3	2.178	2.090
4	1.971	2.004
5	1.695	2.643
6	2.075	2.243
7	2.489	2.067
8	2.195	2.162

**Figure 2:**
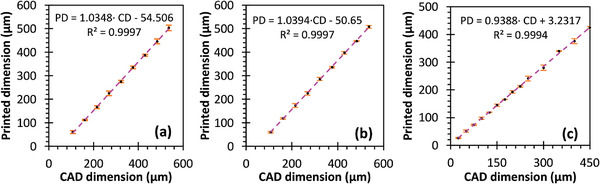
Printed dimension versus CAD dimension in a) X, b) Y, and c) Z axis for objects printed in Solus Proto with an Asiga Max X27. Error bars represent one standard deviation. In all panels, PD = printed dimension, CD = CAD dimension.

**Figure 3:**
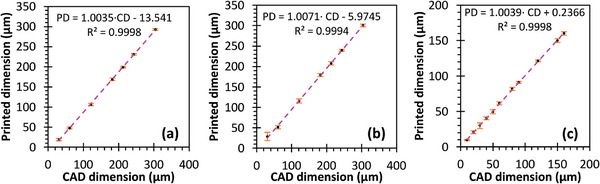
Printed dimension versus CAD dimension in a) X, b) Y, and c) Z axis for objects printed in UpPhoto with a NanoOne 1000. Error bars represent one standard deviation. In all panels, PD = printed dimension, CD = CAD dimension.

Metrology tests were also performed to validate the fidelity and resolution of each 3D printer and printable material. In these experiments, resolution matrices comprising step pyramids with step sizes incrementing by 26.78 µm (one pixel) for the DLP printer and 30.4 µm for the 2PP printer were made. Measurements were taken using a Keyence VK‐X250 laser scanning confocal microscope (Keyence, Itasca, IL, USA), comparing printed dimensions with CAD dimensions. The results exhibited strong linearity, with proportional constants close to 1, and in‐plane offsets of ≈50 µm (≈2 pixels) for the DLP printer (**Figure** **2**) and ≈10 µm for the 2PP printer (**Figure** **3**).

**Figure 4:**
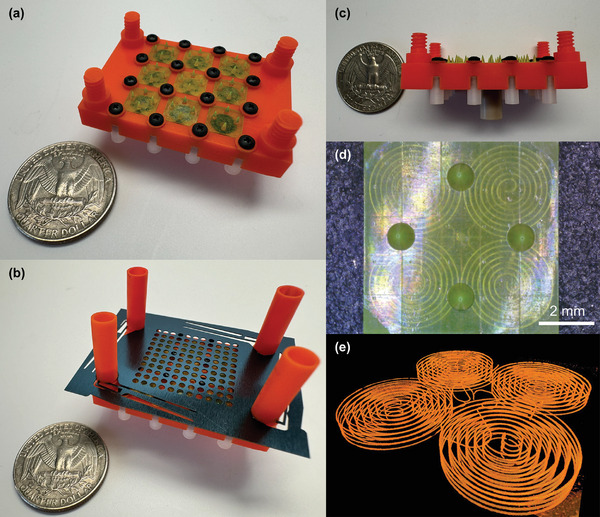
Images of a 3D‐printed electrospray thruster: a) optical image of the device without the extractor electrode next to a US quarter (the central emitter module was replaced with a non‐functional emitter module during the experimental characterization to ensure uniform emitter operation across the other eight emitter modules); b) optical image of a fully assembled device next to a US quarter; c) optical image from the side of the device without the extractor electrode next to a US quarter; d) close‐up optical image of an emitter module; e) X‐ray CT image of an emitter module, evidencing that the hydraulic network inside the module is free from resin.

After printing, both the manifold block and emitter modules underwent a cleaning process to eliminate uncured resin. The cleaning process for the manifold block began with 5 min of ultrasonic cleaning in isopropanol. A fluidic connector, NanoPort Assembly (IDEX Health & Science, Rohnert Park, CA, USA) was then attached to the manifold inlet using super glue. The next day, isopropanol was injected into the manifold block using a syringe pump while being sonicated, ensuring that any remaining resin was removed (Solus Proto is an opaque, orange colored resin; it is straightforward to assess whether the removal of uncured resin is completed). For the emitter modules, a similar process was followed, beginning with a 5 min soak in isopropanol. Afterward, the emitter modules were inserted into the cleaned manifold block, and the inlet of the assembled device was connected to nitrogen pressurized at 6 × 10^5^ Pa (6 bars) for 24 h to purge any residual resin. Following this, isopropanol was again injected into the combined device via a syringe pump while it was sonicated; after that, the system was reconnected to pressurized nitrogen to dry the channels completely. Once the cleaning process was completed, a 5 cm long, 508 µm inner diameter stain‐less steel tube was connected to the manifold inlet to enable electrical connection to the propellant. Finally, the cleaned device was placed inside a vacuum chamber for testing. **Figure** **4**a,c show the manifold block with the emitter modules integrated, while Figure 4b shows the devices with the extractor electrode assembled. Figure 4d shows a close‐up of an emitter module, while Figure 4e shows a 3D image of the cleaned channel obtained using an X‐ray CT scanner, Versa 620 XRM (Zeiss, Oberkochen, Germany).

## 4 Device Characterization

### 4.1 Experimental Setup

**Figure 5:**
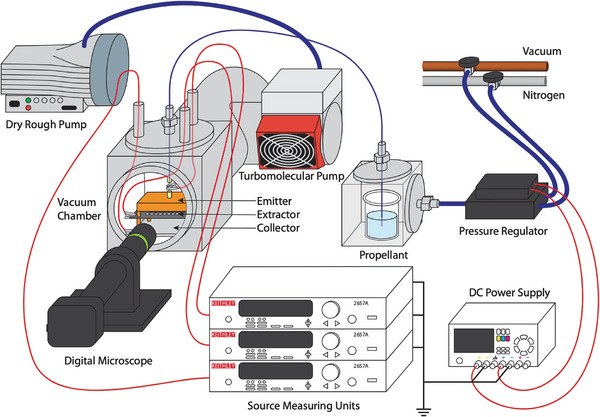
A schematic of experimental apparatus.

The 3D‐printed devices were characterized in a vacuum using a triode configuration (**Figure** **5**). The vacuum chamber (Kurt Lesker, Jefferson Hills, PA, USA) was equipped with a flange with multiple SHV‐5 feedthroughs (Kurt Lesker, Jefferson Hills, PA, USA) and a Swagelok adaptor (Swagelok, Solon, OH, USA) that allowed supplying electrical signals and propellant to the devices. The assembled thruster was mounted over a flat metal plate, which was used as collector electrode. To electrically connect the emitters, a 5 cm long stain‐less steel tube was connected to the manifold block's inlet with fluidic adaptors and used as emitter electrode. The emitter, extractor, and collector electrodes were connected to SHV‐5 feedthroughs using Kapton‐insulated electrical wires with alligator clips. A Keithley source measure unit 2657A (Keithley, Solon, OH, USA) was used for biasing voltages and measuring currents. To avoid electrochemical gas generation at the stain‐less steel tube, the emitter electrode was maintained at 0 V, while the extractor electrode was positively biased.^[^ ^30^ ^]^ The collector electrode was set 200 V above the extractor electrode voltage.

To characterize the devices in a vacuum, the vacuum chamber was first evacuated to below ≈13.3 Pa (0.1 Torr) using a dry rough pump (Edwards Vacuum, Burgess Hill, United Kingdom) and further reduced to ≈1.33 × 10^−4^ Pa (1 × 10^−6^ Torr) using a turbomolecular pump (Pfeiffer Vacuum, Aßlar, Germany). The propellant, EMI‐BF_4_, was supplied through a 508 µm (0.020″) inner diameter PFA tube (IDEX Health & Science, Rohnert Park, CA, USA), with the other end submerged in a propellant vial inside a ConFlat cube (Kurt J Lesker Company, Jefferson Hills, PA, USA). The pressure inside the cube was varied between 0 and 5 × 10^5^ Pa (absolute) (0 and 5 bar) with an uncertainty of 1000 Pa (0.01 bar) using an electronic pressure regulator (Enfield Technologies, Trumbull, CT, USA). To minimize gas bubbles formation at the emitter, the EMI‐BF_4_ reservoir was maintained under a vacuum for at least 24 h to remove any dissolved water.

### 4.2 Emission Stability and Uniformity

**Figure 6:**
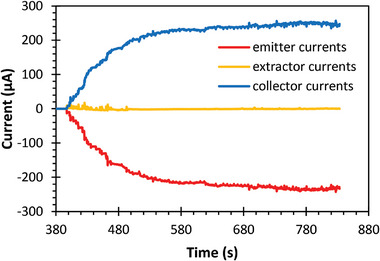
Electrospray array emitter, extractor, and collector currents versus time during startup, eventually reaching stable emission when all emitters are activated.

**Figure** **6** presents the emitter, extractor, and collector currents versus time during the propellant filling‐in phase for a device with 32 emitters (8 emitter modules). In this test, the extractor electrode and collector electrodes were biased at 2000 and 2200 V, respectively, with a non‐functional emitter module positioned at the center of the manifold block for improved flow uniformity. The data show that ≈400 s after pressurizing the propellant tank at 5 × 10^4^ Pa (0.5 bar), EMI‐BF_4_ filled in one channel and initiated the emission. Gradually, other channels were filled in, causing additional emitters to initiate electrospray, increasing the emitter current.

**Figure 7:**
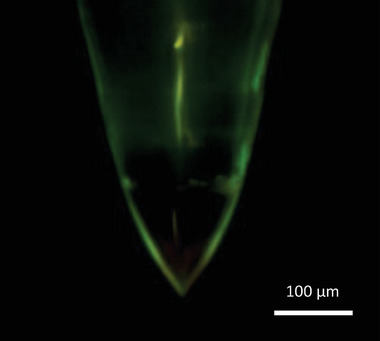
A zoomed in optical image of an emitter tip part of a 32‐emitter array, showing the Taylor cone in stable electrospray emission.

Most of the electrospray beams passed through the extractor apertures, as evidenced by the low extractor current (in the order of hundreds of nanoamperes). The transmitted droplets were collected at the collector electrode, resulting in an inverse current to the emitter. By 700 s, all 32 emitters had formed Taylor cones, which were visually confirmed via a digital microscope (**Figure** **7**). The extractor current stabilized ≈−0.4 µA, ≈600 times smaller than the peak emitter current, indicating a ≈100% electrospray beam transmission.

**Figure 8:**
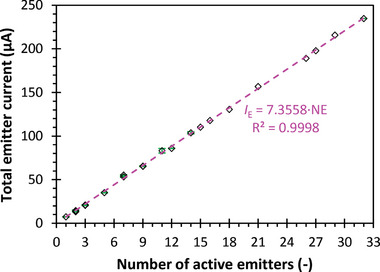
Total emitter current versus number of active emitters from the data shown in Figure 5. Error bars represent one standard deviation. In the figure, ***I***_***E***_ is the total emitter current in µA and ***NE*** is the number of active emitters.

The current data in Figure 6 also validates the uniform operation of the 32 emitters. Between 400 to 700 s, the emitter current showed several locally stable currents that lasted a few seconds between the activation of each emitter. By measuring the current at these locally stable moments, the number of active emitters at a given time was inferred. **Figure** **8** shows a linear increase in emitter current with the number of active emitters, indicating scalable thrust and uniform emitter operation.

### 4.3 Emission Throttlability via Pressure Control

**Figure 9:**
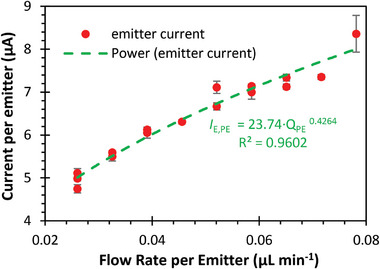
Per‐emitter current versus per‐emitter flow rate for electrospray thrusters with a range of active emitters. Error bars represent one standard deviation.

**Figure 10:**
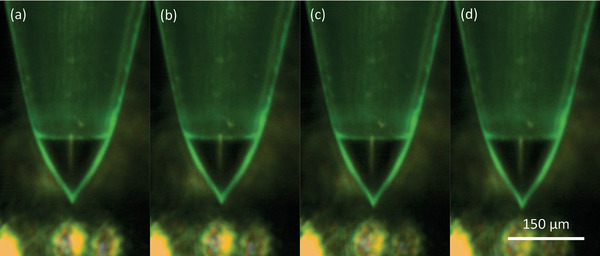
The comparison of the Taylor cone size under different pressure; applied pressures are a) 4 × 10^4^ Pa, b) 6 × 10^4^ Pa, c) 8 × 10^4^ Pa, and d) 1 × 10^5^ Pa (0.4, 0.6, 0.8, and 1 bar, respectively).

Similar tests were conducted with varying numbers of active emitters and different propellant pressure while maintaining the extractor voltage at 2000 V and collector voltage at 2200 V. Once stable Taylor cones formed at all emitters, the emitter current per emitter was measured under different flow rates and plotted in **Figure** **9**. The size of the Taylor cone increases as the pressure applied increases, as shown in **Figure** **10**. A power law fit to the data yielded *I*_*E*,*PE*_ =  23.74 · *Q_PE_* ^0.4264^, where *I*_*E*,*PE*_ is the emitted current per emitter in µA and *Q_PE_* is the flow rate per emitter in µL min^−1^. The observed relationship closely aligns with the well‐known droplet regime current scaling law, which predicts a square root relationship between current and flow rate,^[^ ^31^, ^32^ ^]^ As discussed later, the thrust scales with current, and therefore, this result demonstrates that thrust control is achievable by adjusting the pressure inside the propellant tank.

### 4.4 Emission Throttlability via Voltage Control

**Figure 11:**
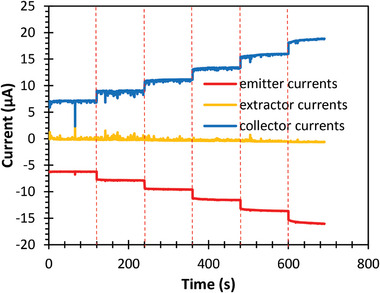
Current versus time for a single‐emitter thruster during stepwise increases in extractor voltage. The extractor voltage was increased by 100 V every 120 s starting at 2000 V, while the collector voltage was biased 200 V higher than the extractor voltage.

**Figure 12:**
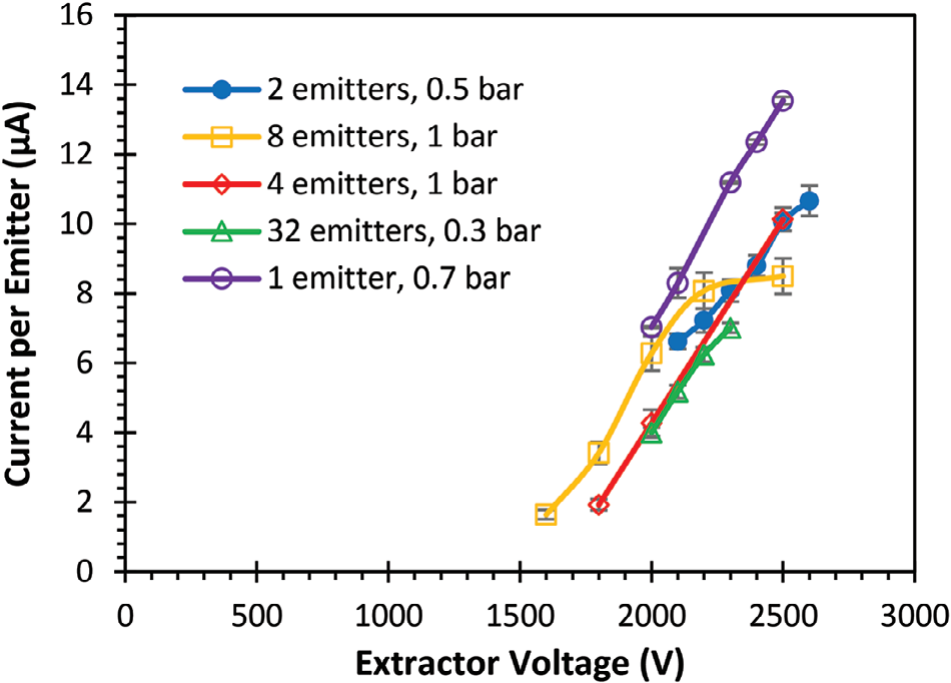
Current per emitter versus extractor voltage for devices with different numbers of active emitters and propellant tank pressures. Error bars represent one standard deviation.

**Figure 13:**
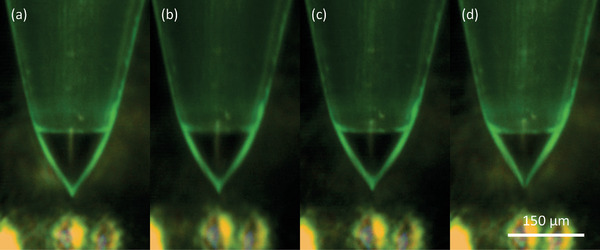
The comparison of the Taylor cone size under different bias voltage; applied voltages on the extractor are a) 1800 V, b) 2000 V, c) 2200 V, and d) 2400 V.

Droplet‐emitting electrospray thrusters typically modulate the emitted current (and consequently thrust) by controlling the flow rate at constant bias voltage. In this study, we demonstrated that it is also feasible to control the emitter current via extractor voltage modulation. **Figure** **11** shows a stepwise increase of emitter current as the extractor voltage was increased by 100 V every 2 min for a single emitter at constant pressure. The collector voltage was also increased by the same amount at each time step. The current reacted to the change in extractor bias voltage immediately and stayed stable at each voltage. Similar tests were done with different number of emitters and pressure, and the results are plotted in **Figure** **12**. Photos taken with a digital microscope show that the size of the Taylor cone decreases as the voltage applied to the extractor increases (**Figure** **13**). Remarkably, the slope of the current versus voltage curves remained nearly constant across varying conditions. Also, despite having a limited voltage range that supports stable electrospray (as can be seen in the data from 8 emitters with 1 × 10^5^ Pa (1 bar) applied pressure in Figure 12), the resulting current and thrust range was greater than what was achieved by controlling the pressure alone.

### 4.5 Thrust and Specific Impulse Estimation

In this study, thrust per emitter *T* and specific impulse *I*_sp_ were estimated by:

(3) $$T=\sqrt{2\dot{m}VI\eta_{T}}$$

(4) $$I_{sp}=T/\left(\dot{m}g\right)$$

where $\dot{m}$ is the per‐emitter mass flow rate of propellant, *V* is the voltage bias between the extractor and the emitter electrodes, *g* is the gravitational constant, and η_*T*_ is the total thrust efficiency, which is the ratio between thrust power and electrical input power. The thrust efficiency can be further divided into several efficiencies:

(5) $$\eta_{T}=\eta_{i}{\eta_{tr}}^{2}\eta_{\theta }\eta_{E}\eta_{p}$$

where η_*i*_ is the ionization efficiency, η_*tr*_ is the transmission efficiency, η_θ_ is the angular efficiency, η_*E*_ is the energy efficiency, and η_*p*_ is the polydispersive efficiency.^[^ ^33^ ^]^ For electrospray thrusters that use EMI‐BF_4_ as the propellant, ionization efficiency is very close to 1 because the low vapor pressure of the liquid suppresses evaporation, which causes emission of neutral material, and the chance of generating electrically neutral droplets in a collision is very low.^[^ ^33^ ^]^ The transmission efficiency is also very close to 1 in the devices reported in this study as can be seen from low extractor current compared to emitter and collector current (see Figure 6). The angular efficiency was estimated from the shape and size of the collected propellant footprint on the collector electrode. From the geometry of it, we estimate a beam half angle of 48°, which yields an angular efficiency of 0.87.^[^ ^33^ ^]^ In the droplet emission mode, the energy efficiency is reduced because of viscous, ohmic, and surface energy dissipation,^[^ ^34^ ^]^ but our calculation suggests that those dissipation mechanisms are negligible for the devices and propellant studied in this work; therefore, the energy efficiency is also close to 1 in the Taylor cone and the jet. The calculation is shown in the appendix. However, there was significant energy loss along the emitter channel given that the emitter voltage was applied at the stain‐less steel tube at the inlet of the manifold block. From the geometry, the electrical resistance of a single channel is *R* = 68.5 MΩ and the resulted energy efficiency is η_*E*_ = (*V* − *IR*)/*V* . The polydispersive efficiency is due to the spread in specific charge of the emitted droplets. This term is hard to estimate without the characterization of the emitted beam, which was not conducted in this study; η_*p*_ =  0.6 was used in this study; the value is the lowest polydispersive efficiency reported for a similar electrospray thruster reported in a different study.^[^ ^17^ ^]^ The estimated thrust and specific impulse are shown in **Table** **3**.

**Table 3:** Flow rate, thrust, and specific impulse for the tests done at constant voltage (0 V biased at the emitter electrode, 2000 V biased at the extractor electrode, and 2200 V biased at the collector electrode). The flow rate, current, and thrust are per emitter values.

Flow rate [µL min^−1^]	Current [µA]	Thrust [µN]	Specific impulse [s]
0.0260	5.11	2.22	404
0.0326	5.59	2.57	375
0.0391	6.12	2.91	352
0.0456	6.31	3.18	331
0.0521	6.67	3.47	316
0.0586	7.00	3.74	303
0.0651	7.34	4.01	292
0.0781	8.36	4.57	278

## 5 Discussion

### 5.1 Voltage‐Controlled Current Mechanism

It has been reported that voltage variation within the allowed range for a single Taylor cone formation per emitter does not significantly impact droplet‐emitting electrospray, as the current is primarily determined by the liquid's flow rate and physical properties.^[^ ^31^ ^]^ However, this was not the case in our experiments, and other studies have also shown significant variations in electrospray current during droplet emission mode when controlled by voltage. For example, a linear voltage‐current relationship was observed for the electrospray of ethylene glycol inside another dielectric liquid, attributed to a relatively small ratio of the emitter diameter to the jet diameter.^[^ ^35^ ^]^ Similarly, a linear voltage‐current relationship was derived for the case when the condition

(6) $$\sqrt{\frac{\rho }{\varepsilon_{0}}}\frac{Q\mathrm{In}\left(2G/D_{0}\right)}{D_{0}\Delta V}\gg 1$$

is not satisfied, where *ΔV* represents the voltage difference between the extractor and emitter tip, ε_0_ is the electrical permittivity of the vacuum, and *ρ* is the mass density of the liquid.^[^ ^36^ ^]^ However, in our experiments this condition was met, and the jet diameter was so small it was invisible through an optical microscope, indicating a large emitter‐to‐jet diameter ratio. Other studies have explored the sensitivity of flow rate to applied voltage, modeling electrical stress at the emitter as a function of voltage,^[^ ^37^, ^38^ ^]^ This was also ruled out in our experiments because the pressure driving the flow was two orders of magnitude higher than the electrical stress.

We speculate there is a different mechanism that enables voltage control in electrospray droplet emission mode, although we are not sure what this mechanism is. Systematic errors in the experiments were ruled out, including leakage through the dielectric (thruster printed body, stand‐offs) between the electrodes or surface contamination. One intriguing finding from our voltage control experiments is the similarity between the slope of the current versus voltage curve (Figure 12) and the reciprocal of the emitter channel's electrical resistance. This suggests that the voltage bias between the extractor and the emitter tip remains mostly constant, regardless of the extractor voltage. While slight changes in this voltage bias may slightly affect the current, they alone are insufficient to explain the observed high current variation, which increased by a factor of 5 or more. It is plausible that increased voltage also raises the flow rate, and we hypothesized that electro‐osmosis flow along the channel may contribute. This could occur because the emitter electrode, located before the microfluidics channel, induces a strong electric field along the channel. However, as discussed in the next section, further experiments showed that electro‐osmosis flow is negligible. Temperature changes were also considered, given that the physical properties of EMI‐BF_4_ depend on temperature, but Figure 11 shows that the current responded almost instantaneously to voltage changes, making continuous heating by electrical current or viscous stress unlikely to cause abrupt shifts. Further investigation, such as measuring the emitter tip voltage and flow rate, or a more detailed study of electrokinetic flow in ionic liquids, is needed to fully understand the voltage‐controlled electrospray current. Although the exact cause is unclear, we speculate that it may be related to the emitter electrode's position upstream of the microfluidic channel, in contrast to most electrospray experiments where the emitters are electrically conductive and directly biased.

### 5.2 Flow Rate Estimation

In our experiments, the flow can be driven by three forces: 1) pressure difference between the inlet and outlet, 2) electrostatic pulling at the emitter tip, and 3) electrostatic forces along the channel (i.e., electrokinetic flow). As mentioned earlier, the electric pressure generated by electrostatic pulling at the emitter tip is two orders of magnitude smaller than the inlet pressure, which is negligible. In terms of electrokinetic flow, both electrophoretic and electro‐osmosis flows are possible. However, electrophoretic flow is negligible as a net flow, given that the anion and cation have similar ionic mobility and cancel each other's movement as a net.

We conducted two additional experiments to assess the impact of electro‐osmosis flow on our results. Electro‐osmosis flow occurs when the channel wall becomes charged from the chemical equilibrium and there is a potential difference across the channel. The charged wall creates an electrical double layer on the liquid side of the interface, with flow induced by the movement of charged particles. The involved chemical equilibrium at the wall is irrelevant to the electric field across the channel, so changing the direction of the electric field also changes the flow direction. Therefore, if electro‐osmosis flow had a large impact on the net flow, changing the voltage polarity should have significantly altered the flow rate and consequently, the current. However, reversing the extractor voltage polarity only changed the current by less than 4%, suggesting that electro‐osmosis flow was not the cause of the current change. Additionally, we conducted a separate experiment with a microfluidic channel printed by a NanoOne 1000 printer using UpPhoto resin. When a voltage difference of 1500 V was applied across a 1 cm long channel containing EMI‐BF_4_, no liquid movement was observed during the 20 min test.

Consequently, we conclude that the flow in our experiments is dominated by the pressure difference between the inlet and outlet. Given that the flow is laminar, with a Reynolds number much smaller than unity, we can estimate the flow rate using the Hagen‐Poiseuille equation. This was verified experimentally when calibrating the experimental apparatus.

### 5.3 Benefits of Voltage Control

While the exact mechanism of voltage control remains unclear, our experiments show that electrospray current scales linearly with applied voltage in the single‐Taylor cone, droplet emission regime. Not all of the applied voltage is used directly in the electrospray due to the voltage drop along the channel, but the resulting current increase enhances thrust. If electrokinetic flow also contributes to an increased flow rate, the thrust would be even greater.

A significant advantage of voltage control is the simplicity it offers in the design of the support and control hardware of the thruster. In systems where the current is solely modulated by flow rate, a control valve with very fine movement is required to throttle the propellant or adjust reservoir pressure, occupying space and adding complexity to the system.^[^ ^39^ ^]^ In contrast, a simple on/off valve is sufficient for voltage‐controlled thrusters, while the added complexity of a power supply that modulates voltage does not significantly add weight or volume. Additionally, despite the limited voltage range that supports stable, single‐Taylor cone electrospray, the resulting current and thrust ranges surpass those achieved by flow rate control alone. Although limited, we were able to apply a wider range of bias voltages to the extractor compared to typical electrospray thrusters because the voltage drop along the channel compensated for changes, maintaining a similar voltage difference between the emitter tip and the extractor. This voltage drop reduces propulsive efficiency, but the resulting high throttlability (up to nearly a threefold increase by adjusting voltage alone) is an attractive trade‐off. The voltage drop also contributes to uniform flow by making each microfluidic channel act as an electrical resistor, promoting current uniformity similar to the use of pre‐resistors individually backing up the emitters in FEEP thrusters to improve array emission homogeneity.^[^ ^40^ ^]^

### 5.4 Thrust and Specific Impulse Comparison

In **Table** **4**, the specifications and performance of our thruster with 32 emitters are compared to the droplet‐emitting electrospray thrusters in the literature. The performance range for our device is based on experiments controlling the flow rate. Propulsive efficiency is not listed, as we only have conservative estimates of our experiments assuming a polydispersive efficiency of 0.6. We expect that the actual thrust and specific impulse will be higher than those shown in Tables 3 and 4, with further testing needed for more precise estimations. Despite this, our device's thrust per emitter is relatively high compared to other droplet‐emitting electrospray thrusters with moderate specific impulse. Furthermore, to the best of our knowledge, ours is the only reported additively manufactured thruster that operates stably in the cone‐jet, droplet emitting regime, offering advantages such as reduced manufacturing cost and compatibility with in‐space manufacturing. Increasing the number and density of emitters is also feasible by adding more emitters to the module and stacking channels vertically. However, we found that the center emitter module received more flow even at low Reynolds numbers and flooded emitters, indicating that some design adjustments are needed to fully utilize the available space.

**Table 4:** Comparison of droplet‐emitting electrospray thruster specifications between this and reported studies.

Study	Number of emitters [‐]	Current per emitter [µA]	Thrust per emitter [µN]	Specific impulse [s]
Hruby et al., 2008^[^ ^16^ ^]^	9	Not reported	0.56–3.98	240–400
Grustan‐Gutierrez et al., 2017^[^ ^17^ ^]^	64	0.132–0.3688	0.13–0.844	96–236
Lenguito et al., 2014^[^ ^19^ ^]^	91	0.310–0.532	0.253–0.716	690–1140
Cisquella‐Serra et al., 2022^[^ ^18^ ^]^	256	0.16–0.29	0.132–0.64	Not reported
Velásquez‐García et al., 2006^[^ ^20^ ^]^	240	0.13–0.28	0.21–2.7	150–350
This Study	32	5.11–8.36	2.22–4.57	278–404

## 6 Conclusion

This study reported a novel, additively manufactured, droplet‐emitting electrospray thruster design that demonstrated scalable and stable emission with competitive thrust performance. The emitter modules were fabricated using a 2PP printer to produce microfluidic channels for uniform flow and small emitters for reduced power consumption. The manifold block, fabricated via DLP, enabled the integration of the smaller emitter modules to scale up thrust. Testing the devices in a vacuum showed that the thrusters achieved thrust between 2.22 and 4.57 µN per emitter and a specific impulse of 280 to 400 s when modulated by pressure, and thrust between 1.89 and 7.25 µN per emitter and a specific impulse of 137 to 400 s when modulated by both voltage and pressure.

Voltage control, in particular, shows significant promise as a method for thrust control, as it eliminates the need for complex valve systems and allows for a wider thrust range. While voltage typically does not affect electrospray current other than triggering the electrospray, our results, similar to findings in other studies, show a linear relationship between current and voltage. However, the mechanism responsible for this in our device differs from previous studies; we hypothesize that the emitter electrode located upstream of the emitter channels may play a role.

Looking ahead, while the proof‐of‐concept demonstration conducted in this study highlights the potential of 3D printing for scalable, droplet‐emitting electrospray thrusters, future research into denser emitter arrays and the mechanisms for voltage‐controlled thrust for these devices would further improve the device's performance. In particular, a more detailed investigation into emitter tip voltage and flow rate, and comparative studies with the location of the emitter electrode, could help clarify how voltage modulation impacts thrust control. With continued development, this technology has the potential to offer a cost‐effective, versatile solution to a wide range of space propulsion applications, particularly in CubeSats and other small satellite platforms, that is compatible with in‐space manufacturing.

## Appendix

### A1 Channel Optimization for Uniform Flow

Two factors contributing to non‐uniformity were considered in this optimization: 1) fabrication tolerances, and 2) the possible range of pressure at the emitter tip. Based on our metrology of resolution matrices using UpPhoto resin printed with a NanoOne 1000 system (using a 10X objective lens, 5 µm layer height, and 4.2 µm in‐plane line distance), we estimate a fabrication tolerance *Δr* = 1.5 µm in channel radius. The pressure at the emitter tip plays a role in the balance of interfacial forces normal to the surface, involving electrostatic stress and surface tension. Given that the electrostatic stress acts outward, the maximum possible pressure at the emitter tip is defined as *p*_max_ = 4γ/*D_o_*  where *D_o_* is the outer diameter of the emitter tip. For the minimum pressure, negative absolute pressure is theoretically possible, but it would result in a concave Taylor cone surface. In our experiments, the Taylor cone surface was always either straight or convex, so we set the minimum pressure, *p*_min_, to zero.

For the optimization, the channel structure is simplified into a single, wider channel that branches into *n* narrower channels. After the junction, each divided channel has a radius *r*_a_ and a length *L*_a_, leading to a hydraulic resistance of *R*_a_ = (8µ*L*_a_)/(π*r_a_* ^4^) . The hydraulic resistance before the junction is *R*_b_. Fabrication errors lead to channel radii ranging from *r*_a –_ *Δr* to *r*_a_ + *Δr*, and the pressure at each channel outlet varies between *p*_min_ and *p*_max_.

In this simplified structure, the worst‐case scenario for flow non‐uniformity is when one channel has a radius of *r*_a –_ *Δr* and an outlet pressure of *p*_max_, while the remaining channels have a radius of *r*_a_ + *Δr* with an outlet pressure of *p*_min_. The flow rate ratio *F_r_* in this case is given by

(A1) $$F_{r}=\frac{R_{a,\max}\left(p_{\mathrm{in}}-p_{\min}\right)+R_{b}\left(p_{\max}-p_{\min}\right)}{R_{a,\min}\left(p_{\mathrm{in}}-p_{\max}\right)-\left(n-1\right)R_{b}\left(p_{\max}-p_{\min}\right)}\;$$

where *R*_a,max_ = (8µ*L*_a_)/(π(*r*_a_ − Δ*r*)^4^) , *R*_a,min_ = (8µ*L*_a_)/(π(*r*_a_ + Δ*r*)^4^) , and *p*_in_ is the inlet pressure. The optimization goal is to minimize *F_r_* with respect to *r*_a_, *L*_a_, and *R*_b_, under the constraint of constant total hydraulic resistance, *R*_b_ + *R*_a_/*n*, for the desired total flow rate.

Given that increasing *L*_a_ always benefits the reduction of flow non‐uniformity, we calculated the optimized values of *r*_a_ and *R*_b_ as functions of *L*_a_. Aiming to achieve uniform flow at low inlet pressure, we set the inlet pressure to 1 × 10^4^ Pa (0.1 bar) and targeted a total flow rate of 0.5 µL min^−1^. Channel length was constraint by the available volume within the emitter module. After several design iterations based on the optimization results, we selected *r*_a_ = 25 µm and *L*_a_ = 0.183 m.

### A2. Energy Efficiency

The energy losses in electrospray droplet emission may be due to viscous, ohmic, and surface energy dissipation. The viscous dissipation, *P*_µ_, and ohmic dissipation, *P*_Ω_, can be estimated based on numerical simulations, which provide the following approximations:

(A2) $$\frac{P_{\mu }}{P_{c}}\approx \frac{12.8+4.53/Re}{{\Pi_{Q}}^{0.23}}$$

(A3) $$\frac{P_{\Omega }}{P_{c}}\approx \frac{7.74{\Pi_{Q}}^{0.39}}{Re^{0.11}}$$

where $P_{c}=\sqrt[{6}]{\left(\gamma^{7}KQ^{3}\right)/\left(\varepsilon_{0}\rho \right)}/\pi$ is the characteristic power, *Re* is the Reynold number defined as $Re=\sqrt[{3}]{\varepsilon_{0}\rho \gamma^{2}/K}/\mu$, Π_*Q*_ is the nondimensional flow rate given by Π_*Q*_ = (ρ*KQ*)/(ε_0_γ)  and *µ*, *γ*, and *K* are the viscosity, surface tension, and electrical conductivity of the propellant at 25 °C, respectively.^[^ ^41^ ^]^ While these approximations do not account for the dependency on the dielectric constant ε of the propellant, the dielectric constant of EMI‐BF_4_ falls within the ranged used in the referenced study, making these approximations applicable in this context.

The surface energy dissipation can be calculated based on the power required to generate the surface area of emitted droplets. Assuming monodisperse droplets, this dissipation is approximated by

(A4) $$P_{\gamma }\approx \frac{3\gamma Q}{r_{D}}$$

where *r_D_* is the radius of the droplet. The droplet radius can be further estimated using the empirical relationship.^[^ ^32^ ^]^

(A5) $$r_{D}=\frac{1}{2}\;{\left(\frac{\varepsilon_{0}\rho Q^{3}}{\gamma K}\right)}^{1/6}$$

Using these approximations, the energy efficiency η_*E*_ can be determined by the voltage deficit, i.e.,

(A6) $$\eta_{E}=1-\frac{\left(P_{\mu }+P_{\Omega }+P_{\gamma }\right)/I}{V}$$

Based on the physical properties of EMI‐BF_4_ at 25 °C (ρ = 1290 kg m^−3^,^[^ ^42^ ^]^ *γ* = 0.0443 N m^−1^,^[^ ^42^ ^]^ *µ* = 0.038 Pa s,^[^ ^42^ ^]^ *K* = 1.36 Si m^−1^,^[^ ^43^ ^]^ and *ε* = 12.8),^[^ ^44^ ^]^ the calculated energy efficiency is ≈0.993, indicating that energy dissipation is negligible.

## Conflict of Interest

The authors declare no conflict of interest.

## Data Availability

The data that support the findings of this study are available from the corresponding author upon reasonable request.
